# Elderly and their companion animals, cause for pleasure or for harm, a case report of a non-bite *Pasteurella multocida* bacteremia in an 85-year-old woman with a fatal outcome

**DOI:** 10.1186/s12877-023-04224-2

**Published:** 2023-09-01

**Authors:** Evien Ali, Niek Tytgat, Lieven Vergote, Katleen Devue, Bart Nonneman

**Affiliations:** 1 Department of Emergency Medicine, UZ Leuven, Leuven, Belgium; 2 Department of Intensive care, UZ Brussels, Jette, Belgium; 3 Department of Intensive care, ASZ, Aalst, Belgium; 4 Department of Emergency Medicine, ASZ, Aalst, Belgium

**Keywords:** *Pasteurella multocida*, Zoonosis, Non-bite *Pasteurella multocida* bacteremia, Case report

## Abstract

We present a case of an elderly woman suffering from *Pasteurella multocida* (*P. multocida*) bacteremia, without obvious bite marks, but owning a pet dog. Although the patient was not immunocompromised, and prompt treatment with intravenous antibiotics was initiated, she developed severe septic shock with multiple organ failure and died. In healthy individuals, an infection caused by these bacteria is easy to treat and often harmless, whereas in elderly serious complications can occur. We believe this report of a fatal outcome of a *P. multocida* bacteremia in a non-immunocompromised, but elderly patient is important, given the growing cohort of elderly pet-owners seeking medical care. A greater awareness of this zoonosis and its potentially fatal outcome is warranted.

## Introduction

90 million households in the European Union own a pet, often living in close contact with the owners [1]. Companion animals can transmit potential pathogens to humans, and especially elderly people (> 65 years) are more susceptible to the risk of pet-induced zoonosis [2, 3]. The benefits in this age group from having a pet include the positive influence it has on mental and physical health [4], such as decreased feelings of loneliness and anxiety [5]. Research showed that older pet owners are less concerned about the risk of zoonotic transmission of disease [6], yet they are more susceptible to health problems occurring after infection. We present a case of an 85-year-old dog owner who got infected with *P. multocida* that caused sepsis with lethal outcome.

## Case presentation

An 85-year-old woman was brought to the emergency department, after being found on the floor by her family, confused and with a fever. History taking was difficult. Since the patient lived alone, no further information could be retrieved. Her medical history was quite brief, with diabetes mellitus type 2, a Trans catheter Aortic Valve Implantation (TAVI) performed 4 years earlier, and hypertension.

The initial clinical observations showed a dehydrated patient with decreased turgor, a normal heart and lung auscultation, a hematoma on the left side of the abdomen with normal palpation of the abdomen. More noticeable was oedema and erythema of the right lower leg, tender on palpation, without any scratch or bite marks. There was no neurological deficit. The patient had a normal blood pressure of 141/75mmHg, a regular heart rate at around 79 bpm, 95% saturation on room air and fever with a temperature of 39.5 °C. Blood count showed a discrete anaemia with haemoglobin of 10.5 g/dL, leukopenia (1700 /mm³) with a normal distribution and high C-reactive protein (CRP) 212.5 mg/L (normal < 5 mg/L), acute kidney deficiency (creatinine 1.61 mg/dL, GFR 30mL/min), normal electrolytes, normal liver enzymes, but discretely elevated creatine kinase levels of 428U/L (normal < 167U/L) and elevated NT-proBNP of 28.500 pg/mL (normal < 500pg/mL). Urinary count showed no signs of a urinary tract infection. X-rays of the pelvis, the right knee and lower leg showed bilateral hip prosthesis and a prosthesis of the right knee, but no fractures or other pathological conditions were found. The X-ray of the thorax showed the known aortic valve prosthesis, but no signs of pulmonary infection or pulmonary oedema. A cerebral Computer Tomography (CT)-scan was negative for any posttraumatic injuries, nonetheless there were some old ischaemic lesions in the right temporal lobe. The patient was admitted to the geriatric ward with a tentative diagnosis of erysipelas of the right lower leg. Treatment included empirical intravenous antibiotics (amoxicillin-clavulanic acid 4 times 1 gram intravenously given daily), Low Molecular Weight Heparin (LMWH), IV fluids, and application of cold compresses.

On the first day of admission, the patient became hypotensive while complaining of abdominal discomfort. An abdominal CT scan revealed no clear signs of infection, but showed bilateral pleural effusions, indicative of heart failure. A new blood count showed thrombocytopenia (43.000/mm³), deterioration of the kidney function (creatinine 2.43 mg/dL and GFR 19mL/min) and an increase of the CRP (298.8 mg/L). Due to progressive sepsis and the development of septic shock, the patient was admitted on the ICU and amikacin was added to the antimicrobial treatment.

In the next few days, the patient developed anuria, a severe thrombocytopenia (6000/mm³) and spontaneous bruising. Differential diagnosis included idiopathic thrombocytopenia, disseminated intravascular coagulation, and heparin-induced thrombocytopenia. LMWH treatment was stopped, and platelet transfusion was started. Extensive thrombocytopenia was most likely a result of the severe septic shock and increased platelet consumption. Transthoracic cardiac ultrasound showed an ejection fraction of 40–50% with right heart overload, but no evidence of endocarditis.

Blood cultures showed growth of *P. multocida*, confirming the hypothesis of an erysipelas-provoked sepsis. As a result, amikacin treatment was stopped. Information from family members revealed that the patient owned a dog, a known carrier of the *P. multocida.* The infection was likely acquired from the pet dog.

The final results of the antibiogram showed a multi-sensitive *P. multocida*. Despite the sensitivity of this species for amoxicillin-clavulanic acid, the patient was escalated to piperacillin-tazobactam (4 times 4 g intravenously given daily) given the rapid deterioration.

Neither the antibiotic therapy, nor added corticoid therapy, platelet transfusion, inotropic treatment with dobutamine or fluid resuscitation could ameliorate the overall state of the patient. In the following days, her condition deteriorated further with persistent oliguria, development of refractory lactic acidosis, recurrent hypoglycemia and finally multiple organ failure. In consensus with the patient’s family and taking into consideration her age, frailty and poor prognosis, the multi-disciplinary decision to initiate a palliative comfort treatment was taken and the patient died the following day.

## Discussion

The *Pasteurella* genus was first isolated by Louis Pasteur in 1880 and named in his honour [7]. *Pasteurella* species have been linked to many infectious diseases in humans and animals. The *P. multocida* subspecies is often found in domestic pets such as dogs and cats, but also in birds who harbour it in their normal upper respiratory microbiota [8]. It’s a small, non-motile anaerobic Gram-negative penicillin-sensitive coccobacillus, and causes an opportunistic infection in humans after bites or scratches [8]. It is responsible for infection in 50% of dog bites and up to 75% of cat bites [2, 9, 10]. In our case, there was no evidence of an animal bite, an observation that has also been described in literature [11]. Studies showed that patients who suffer from a *P. multocida* infection without a prior animal bite, are more likely to be immunocompromised of suffer from severe comorbidities. These patients show longer hospital stays, more systemic infections, a higher chance to be hospitalised at the ICU, and a higher mortality rate [10].

P. multocida has been associated with a wide range of infection syndromes, including septic arthritis, necrotising fasciitis, osteomyelitis, septic shock, meningitis, endocarditis and severe respiratory tract infections [12–16]. Infections of prosthetic material, dialysis catheters or long-term central venous lines are rare but require our attention [17].

Mortality rates of *P. multocida* bloodstream infection have been found to be up to 30% [18], although other retrospective studies in literature have shown a lower mortality rate as low as 8% [19].

Therefore, the importance of prophylactic antibiotic therapy is not up for discussion, and a combination of amoxicillin and β-lactamase inhibitor clavulanic acid is preferred. In patients with penicillin allergy, the combination of clindamycin and a fluoroquinolone, such as ciprofloxacin, is recommended [20].

In this case the most probable entry point was a minor scratch by a domestic pet, and even though our patient was not severely immunocompromised, we saw a rapid deterioration resulting in death. This left us incapable to further investigate a possible infection of the prosthetic aortic valve or the bilateral hip and right knee prothesis.

In non-bite patients a good clinical evaluation and history taking is crucial, yet this presents a challenge, especially in an elderly population. The present case warrants a more aggressive treatment recommendation for this growing age group.

## Conclusion

In healthy, non-immunocompromised patients, an animal bite is often a minor inconvenience without serious consequences. Treatment is easy and recovery from an infection occurs mostly without complications. This is however not the case in an immunocompromised population or in patients with severe comorbidities, which elderly often are. Here, morbidity and mortality rates for *P. multocida* bacteremia are a lot higher, opposed to the healthy population. The present cases issues a warning not to forget about this type of entry point for bacteria, and to engage in a thorough history taking in the elderly patient. The history should also include the presence of pet companions in the home setting.

## Data Availability

All data generated or analyzed during this study are included in this published article.

## Abbreviations

*P. multocida*: *Pasteurella multocida*
TAVI: Trans catheter Aortic Valve Implantation
CRP: C-reactive protein
GFR: glomerular filtration ratio
CT scan: computed tomography scan
